# Extensive Ossification of the Achilles Tendon with and without Acute Fracture: A Scoping Review

**DOI:** 10.3390/jcm10163480

**Published:** 2021-08-06

**Authors:** Daniel Sullivan, Allison Pabich, Ryan Enslow, Avery Roe, Donald Borchert, Keenan Barr, Bailey Cook, Amanda Brooks

**Affiliations:** College of Osteopathic Medicine, Rocky Vista University, Ivins, UT 84738, USA; allison.pabich@rvu.edu (A.P.); ryan.enslow@rvu.edu (R.E.); avery.roe@rvu.edu (A.R.); donald.borchert@rvu.edu (D.B.); keenan.barr@rvu.edu (K.B.); bailey.cook@rvu.edu (B.C.); a.brooks@rvu.edu (A.B.)

**Keywords:** Achilles Tendon, Extensive Ossification, heterotopic ossification, Achilles Tendinopathy, Calcific Tendinopathy

## Abstract

Extensive Ossification of the Achilles Tendon (EOAT) is an uncommon condition characterized by the presence of heterotopic ossification within the substance of the Achilles Tendon and is distinct from other tendinopathies associated with tendon mineralization. The purpose of this scoping review of the literature on EOAT is to describe the pathogenesis, patient population, presentation, management, and outcomes of this rare condition. Fifty-four articles were included in the scoping review after screening and selection. According to the literature, EOAT often presents with pain and swelling around the Achilles Tendon and is frequently associated with acute trauma. EOAT is more common in men, and although the exact mechanisms of the pathology are not fully understood, EOAT may demonstrate specific molecular signaling patterns. The lack of knowledge regarding the molecular mechanism may be a significant hindrance to the management of the condition. Even though a standard treatment regimen for EOAT does not exist, conservative management for six months in patients without complications is recommended. Those who have an acute fracture of the ossification should be managed more aggressively and will often require surgical repair with autograft, although there is no standardized procedure at this time. Clinicians should be aware of the typical presentation, risk factors, and management options of patients with EOAT. Additionally, they should be cautious when selecting treatment strategies and conduct a thorough evaluation of long-term outcomes with various treatment modalities, which this review provides. Most important, this review highlights the need for further research to determine the best course of clinical treatment of EOAT injuries, in order to establish a standard treatment regimen.

## 1. Introduction

Achilles Tendinopathy and related conditions often result from a failed healing response to chronic tendon injury [1]. Achilles Tendinopathy is characterized by pain, swelling, and limited function of the tendon, and may include calcification or ossification [2]. Regardless of the specific pattern, several terms are used to describe calcification/ossification within or surrounding the Achilles Tendon, including Heterotopic Ossification of the Achilles Tendon, Calcifying Tendonitis, Ossified Tendonitis, Calcific Periarthritis, and Apatite Deposition [2,3]. In contrast to Calcific Tendinitis, Ossific Tendinitis microscopic examination reveals one or more segments of mature lamellar bone formation within the substance of the tendon, either at the insertion or within the body of the tendon [4,5,6]. Rarely, the Achilles Tendon completely or partially ossifies over time. We refer to this condition as Extensive Ossification of the Achilles Tendon. In this review, “extensive” is defined as ossification within or surrounding the Achilles Tendon that comprises at least roughly one-third of the affected tendon.

Extensive Ossification of the Achilles Tendon differs from other types of Achilles tendinopathies in several ways. First, many individuals with EOAT report a history of acute trauma or surgery to the Achilles, often decades before [2]. This is in contrast to other types of tendinopathy which tend to occur after chronic degeneration. Second, EOAT may go unnoticed for decades until the heterotopic bone fractures [3,5]. Third, because EOAT is rare, little research has been conducted to identify the pathophysiology, risk factors, treatments used, and outcomes of these treatment approaches.

Despite the lack of a defined pathophysiology, an important distinguishing factor of EOAT is that it may cause the ossified Achilles to fracture after decades of being silent, which often presents with acute tenderness, pain, localized edema, and decreased range of motion. Diagnosis is confirmed with plain radiographs and is characterized by the presence of one or more segments of ossified masses of variable sizes within the body of the Achilles Tendon [7].

When Achilles Tendon radiopacities are identified on plain radiographs, they are characterized as falling into one of four types. Type I refers to radiopacities at the tendon insertion. Type II refers to radiopacities found 1–3 cm proximal to tendon insertion, and Type III refers to larger, more proximal radiopacities. Type III ossifications are further divided into Type IIIa and Type IIIb. Type IIIa is characterized by partial ossification. Type IIIb indicates complete ossification of the tendon [8,9,10]. The diagnosis can be further delineated with ultrasound or magnetic resonance imaging [11].

Various treatments for Achilles Tendinopathy, Calcific Tendinopathy, and Insertional Calcific Tendinopathy have been studied extensively [1]. Conservative management strategies that have been utilized include eccentric exercise techniques, orthotics, medications such as non-steroidal anti-inflammatory drugs (NSAIDs) and nitric oxide patches, shock wave therapy, ultrasound therapy, and various types of locally injected medication. Multiple surgical treatment approaches are also commonly utilized [1]. These include removal of the ossified Achilles and replacing it with an autograft from tendons of the semitendinosus, gracilis, fascia lata, gastrocnemius-soleus muscle, and flexor hallicus longus [5,12,13,14,15].

This review focuses on Extensive Ossification and excludes minor Achilles calcification. Achilles Tendon ossification has been classified in numerous ways in order to assist in identification of site, severity, and type. Here, we discuss the various treatment approaches that have been reported in the literature as well as the outcomes associated with each treatment in order to identify and summarize viable treatment strategies aimed to improve patient outcomes.

## 2. Materials and Methods

This scoping review was conducted from November 2020 to May 2021. The current review method followed the methodological framework described by Arksey and O’Malley, who defined a scoping review as a way to “map rapidly” key concepts of literature on selected research topics and provide information on the main types of evidence published [16]. A scoping review allows for efficient summarization and dissemination of current literature and enables readers to quickly identify gaps in the literature. It should be made abundantly clear that a scoping review does not appraise the quality of literature, which is a limitation that should be considered with this type of review.

### 2.1. Research Question

To understand and describe the pathogenic mechanisms, patient population, risk factors, presentation patterns, reported treatments, and outcomes of each treatment approach for treating Extensive Ossification of the Achilles Tendon.

### 2.2. Identifying Relevant Research

**Figure 1:**
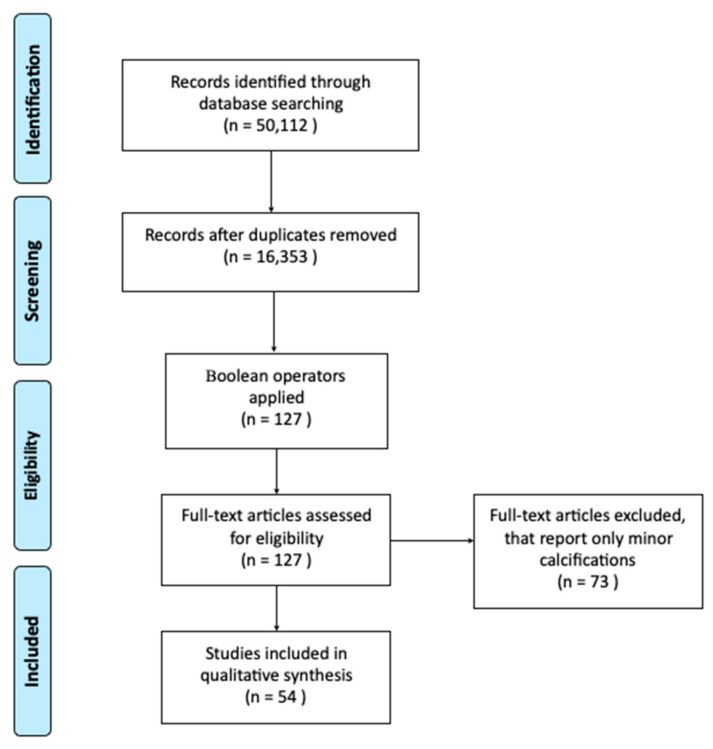
Flowchart for identification of relevant research, screening and selection. Based on PRISMA guidelines and the scoping review framework described by Arksey and O’Malley [16].

Studies that describe Class I, II, IIIa, and IIIb Achilles Tendon ossification regardless of whether or not the patients presented with a fracture of the ossification were collected (Figure 1). All relevant literature in the following categories were reviewed: basic science and molecular science studies, case reports and case series, clinical studies, clinical images, and literature reviews. Publications between the dates of November 1932 and May 2021 were reviewed. An exhaustive search was conducted using PubMed (National Library of Medicine, Bethesda, MD, USA), MEDLINE (Medline Industries Inc., Northfield, IL, USA), ClinicalKey (Elsevier, Amsterdam, The Netherlands), the Directory of Open Access Journals (Infrastructure Services of Open Access C.I.C., Devon, UK), and Cochrane databases (Cochrane, London, UK). The search terms used to find the related literature from all databases included: “Achilles calcification,” “endochondral calcification,” “Achilles ossification,” “calcification,” “acute fracture,” “calcific tendinitis,” “Extensive Ossification,” and “extensive calcification.” These search terms alone revealed a list of 50,112 articles. The list was then narrowed using Boolean operators in coordination with search tools for each database which yielded 127 total articles. Authors then reviewed each article and included 54 out of the 127 for this scoping review. Although the two terms “ossification” and “calcification” of the Achilles Tendon are distinct pathologies, the terminology has been erroneously used synonymously in the literature, therefore the latter was included in the search terms for completeness.

### 2.3. Screening and Selection

Case reports and clinical images were only selected if they included Extensive Ossification of the Achilles Tendon. Literature reporting minor calcifications were excluded from the review, as their presence within the Achilles Tendon is described extensively in other literature. Furthermore, they are often asymptomatic and clinically insignificant unless they are bothersome to the patient. Alternatively, Extensive Ossification presents with unique features such as an acute presentation of pain with or without an acute fracture and limited range of motion. Basic and molecular science literature evaluating the pathogenesis of tendon calcification was included whether or not the contents were specific to Extensive Ossification of the Achilles. In addition, literature reviews and clinical studies were included if they discussed Achilles Tendon calcification and ossification whether or not they were specific to Extensive Ossification of the Achilles. Case reports and clinical images that contained minor calcification were excluded. All literature that reported Extensive Ossification whether or not an acute fracture of the ossification was present was included. Literature was not excluded based on different treatment options or outcome.

## 3. Results

In total, 54 research articles were included in this scoping review. There were 25 case reports (Table 1) and five clinical images with an associated case description (Table 2) that were included. Table 3 includes a list of each case report and clinical image with check marks to indicate presence of certain features of the case, including the sex of the patient, whether the ossification was fractured, whether conservative or surgical treatment was used, and whether or not there was a history of acute trauma to the tendon. Authors also identified and included four case series (Table 4), 10 molecular studies (Table 5), two clinical studies (Table 6), and eight review articles (Table 7). Table 4, Table 5, Table 6 and Table 7 have been included to provide summaries of the relevant findings of the aforementioned studies. The majority of the patients (19 out of 30) who were described in the case reports and clinical images included in this scoping review are men, which is in agreement with previous findings that Achilles ossification affects men twice as much as women [17].

As reported in previous studies, Achilles Tendon ossification often results from previous trauma, tendon rupture, or surgery involving the Achilles Tendon. Of the 30 case reports and clinical images analyzed, 16 reported a history of acute trauma to the ankle or Achilles Tendon, four had a history of surgery for clubfoot release, 19 presented with a fracture of the ossification, 12 were treated conservatively, and 18 underwent surgical repair. Other posited triggers for ossification that have been described include Wilson’s disease, Reiter’s syndrome, gout, metabolic disease, and repetitive trauma [9,37]. Alternatively, Cortbauoi et al. presented a case series of three siblings, all without history of predisposing factors, who each developed ossification of the Achilles Tendon, suggesting a possible element of genetic predisposition [37] (Table 3).

Numerous molecular studies included in this scoping review demonstrated molecular processes that occur with Achilles Tendon ossification. The normal healing process of tendons and ligaments alike involves a chronologically staged and regulated process of inflammation, proliferation, and remodeling [56] (Table 4). Each stage of the process is characterized by distinct cytokines and cellular processes. The inflammatory stage begins immediately after the injury and is characterized by release of TGF-B, IGF-1 and PDGF from clotting cells. These events recruit neutrophils, which activate macrophages to clear debris caused by the injury [56]. The proliferation stage begins around day two post-injury, where cytokines, namely TGF-B and IGF-1, stimulate production of collagen and other extracellular matrix (ECM) components. These cytokines both help recruit fibroblasts and platelets that facilitate the formation of a fibrovascular scar at the site of injury [56]. From the second to third week, the remodeling process begins. During the proliferative phase of the remodeling process, fibroblasts secrete type III collagen which is relatively weak and prone to injury. Two weeks after injury, tenocytes deposit type I collagen arranged in fibrils, which increases mechanical strength. This process is known as fibrilo-genesis. Fibrils then bind together in end-to-end and lateral directions, which further increases the tensile strength of the tissue. The last phase of wound healing is known as the remodeling phase. This phase begins at 3–6 weeks after the injury and may continue up to 1 year after the injury. During this time, the damaged tissue slowly decreases in vascularity, number of cells, and collagen fiber density. There is also an increase in elasticity and strength of the tissue during this phase [56].

Due to the complex signaling pathways and lengthy duration of tendon healing, there are multiple pathways that have been explored as an underlying mechanism in the formation of tendon ossification. Zhang et al. demonstrated that TGF-B is likely to contribute to calcification of tendons by using a nuclear retinoic receptor gamma agonist to inhibit mineralization mediated through the BMP-Smad pathway [45]. This makes sense considering that Zhang et al. also identified bone morphogenic protein-Smad (BMP-Smad) signaling as a potential ossification pathway. The BMP protein, which is part of the Transforming growth factor-beta (TGF-B) superfamily, is a signaling molecule that has been shown to play a role in bone formation. Zhang et al. also showed that treatment with a BMP receptor kinase inhibitor significantly reduced tendon mineralization. Wang et al. confirmed these results by showing that heterotopic ossification progression can be prevented through inhibition of TGF-B using both antibodies and gene knockout strategies [45,46]. Similarly, Tuzmen et al. showed that Substance P induced heterotopic ossification, and blocking substance P with calcitonin gene-related peptide inhibited heterotopic ossification [47]. Investigators have also alluded to the importance of cellular processes. Agarwal et al. showed that bone-cartilage-stromal progenitor cells are enriched at sites of heterotopic ossification [44]. Although the mechanism of tendon ossification is not fully understood, these molecular studies point toward the possibility of targeted drug or biologic treatments to treat or prevent Extensive Ossification of the Achilles Tendon.

Table 5 includes summaries of two clinical studies assessing the incidence of Achilles Tendon calcification after surgery, further demonstrating that surgery is a common source of trauma that leads to calcification and perhaps eventually to ossification. Beyond potential targeted pharmaceutical approaches based on the biochemistry of bone formation, treatment options for an ossified Achilles Tendon consist of either conservative or surgical options. The decision about which treatment approach to initiate is typically determined by factors such as pain, the extent of ossification, and whether the ossified segment of tendon has fractured. When no fracture or pain is present, conservative methods such as equinus below-knee casting has been described in several reports. Haddad et al., Marino et al., Parton et al., and Banerjee et al. all showed positive results following conservative treatment through cast immobilization [8,25,27,28]. However, with extensive calcification of the ossified fragments, or when non-operative therapy has been unsuccessful for roughly six months, surgical excision of the calcified masses and repair of the Achilles Tendon has been favored in several reports [1,38]. Howell et al. and Proctor et al. demonstrated that surgical intervention is an effective treatment for an ossified Achilles Tendon following failed conservative treatment [20,38]. Hatori et al. and Sasaki et al. also showed positive results with surgical removal of an ossified mass [30,36].

Out of the 19 case reports and clinical images that reported a fracture of the ossification, 13 were treated surgically. Out of the 11 reports that described a patient without a fracture, five were treated surgically. There have been numerous methods described in case reports which are included in this scoping review. Brotherton et al. and Mallinson et al. describe a technique wherein the ends of a fractured tendon can be brought and held together with a wire [18,19]. However, more commonly, the ossified segments of the Achilles are removed, with the remaining tendon ends reconnected with a tendon graft [4,5,10,13,14,15]. Tendons from the flexor hallucis longus, hamstring, semitendinosus, gracilis, fascia lata, plantaris, and fascia overlying gastrocnemius-soleus muscle have all reportedly been used as tendon autografts [5,12,13,15] with no allografts or xenografts being used in the reports.

## 4. Discussion

Ossification of the Achilles Tendon is an uncommon condition with an unknown prevalence. There are a limited number of studies, most of which are case reports of patients who present with an acute fracture of the ossified mass. Other reports describe patients without a fracture who have a large ossification and presented with pain and swelling. Since many ossified Achilles Tendons are silent for decades and may or may not result in an acute fracture of the ossification, many patients likely are asymptomatic and have undetected ossification.

### 4.1. Presentation

Extensive Ossification of the Achilles Tendon usually presents decades after an acute traumatic event to the lower leg. A common traumatic event is lower leg surgery during childhood. However, in some cases, there is no attributable prior traumatic event. Patients who present with a fracture to the ossified Achilles Tendon most commonly describe acute pain and swelling of the lower leg and occasionally an audible “pop.” The condition is approximately twice as common in men [42]. There is no current explanation for the male preponderance, but hormones and varying activity levels should be considered [42]. The mean age of presentation, calculated from our collection of 30 case reports and clinical images, is 55 years old with a range of 24 to 84 years old [20,31]. On physical examination, the ossified Achilles Tendon may have an indurated feel, with some reports suggesting the presence of a palpable gap at the site of the fracture. The mass is typically not tender or painful until the ossification fractures or the Achilles Tendon ruptures. Depending on whether the Achilles Tendon is intact or not, the patient may have a positive Thompson test [4]. Extensive Ossification of the Achilles Tendon may also present without an associated fracture. In cases of Extensive Ossification without an acute fracture, patients often present with insidious pain and swelling to the affected heel, restricted range of motion, and limitations in activities of daily living [30,37].

### 4.2. Diagnostic Criteria

When providers suspect the presence of EOAT, the first imaging modality that should be performed is plain radiographs or in-office ultrasound if available. In-office ultrasound will likely reveal the hyperechoic mass and may detect a fracture if present [35]. Plain radiographs should be obtained to view the extent of the ossification and whether there is a fracture present [7,11,34]. Plain films are also used to categorize the ossification based on the location of ossification in the Achilles Tendon. The Morris classification, which classifies Achilles calcification/ossification based on the distance from the insertion of the tendon onto the calcaneus, is sometimes used. Additionally, obtaining plain film radiography soon after the initial office visit will provide a baseline upon which future imaging can be used to compare. Although Magnetic Resonance Imaging (MRI) is not required to make the diagnosis of EOAT [31], it can be used as an adjunctive diagnostic method to assess the integrity of the tendon, providing more detail regarding the composition and location of the ossification and the extent of soft tissue destruction. Magnetic Resonance Imaging is also useful in identifying a hematoma [2].

### 4.3. Histology

Calcification and ossification are two distinct forms of tendon mineralization. Calcium that deposits in normal, healthy tissue is called metastatic calcification. Calcium that deposits in damaged tissue is called dystrophic calcification. In calcific tendinitis, the tissue contains calcium phosphate and/or calcium carbonate, which is not organized into an orderly structure microscopically. Additionally, the calcification in calcific tendinitis may regress over time as part of healing [57]. In contrast, the term ‘ossification’ suggests the formation of bone [58]. True ossific tendonitis will demonstrate crystals of hydroxyapatite, which are organized into lamellar bone [4,5]. Heterotopic bone formation usually occurs via endochondral ossification [42]. Both calcific tendonitis and ossific tendonitis are more common in men. Achilles Tendon ossification is much rarer than Achilles calcific tendonitis [5].

### 4.4. Pathobiology and Risk Factors

Heterotopic ossification, defined as bone developing de novo in soft tissue, may be a consequence of degenerative changes in collagen caused by vascular insufficiency. Vascular insufficiency leads to chronic tissue hypoxia, leading to an inflammatory cascade that causes mesenchymal stem cells in the tendon to differentiate towards bone development [2,27,30,59]. Up to 62% of patients who undergo percutaneous or open repair of the Achilles Tendon develop mineralization, but the proportion of cases which progress to Extensive Ossification is unknown [49].

The etiology of Achilles Tendon ossification is likely to be multifactorial and may be a result of overt trauma, repetitive micro trauma, surgery, or burns to the Achilles Tendon [36,44,59]. Some systemic diseases have been seen in conjunction with ossification, including diffuse idiopathic skeletal hyperostosis, fluorosis, ochronosis, Wilson’s disease, renal failure Reiter’s syndrome, ankylosing spondylitis and gout, although these conditions have not been proven as causative [27,59]. In the reports of EOAT, there were no abnormalities in the bloodwork that would suggest presence of the associated disorders.

Despite no recognized abnormalities in routine bloodwork, there may be a genetic predisposition, as ossification of the Achilles has been reported in biological siblings without history of trauma or surgery to the tendon. The age of presentation of the siblings were 50, 54, and 64 years old [37]. This indicates that, if there is a hereditary component, the age of onset is consistent with most cases that are reported, which have an average age of presentation of 55 years old. Furthermore, two rare inherited conditions that cause heterotopic bone formation include fibro-dysplasia ossificans progressiva and progressive osseous hetero-plasia. Fibro-dysplasia ossificans is caused by a mutation of Activin receptor type-1 (ACVR1), which is a bone morphogenetic protein (BMP) type I receptor. The mutation causes overstimulated BMP signaling, leading to inappropriate ossification [60]. Progressive osseous hetero-plasia (POH) is an inherited condition caused by an inactivating mutation in the GNAS locus, which leads to inappropriate ossification of the dermis and adipose tissue [60].

Genetic predisposition to tendon calcification/ossification has also been studied in murine models, and there is evidence suggesting that TFG-B/BMP superfamily protein upregulation, BMP type1 receptor mutations, hypoxia inducible transcription factor (HIF), and bone-cartilage-stromal progenitor (BCSP) phenotype may contribute to endochondral ossification, which is the most frequent ossification pattern documented [42,44,45]. The underlying pathophysiology of fibro-dysplasia ossificans progressiva and progressive osseous heterotopia, along with other molecular studies demonstrating the role of cell signaling pathways, suggest that heterotopic ossification is likely to be due to dysregulation of certain signaling pathways. The dysregulation may be induced by acute or chronic trauma, genetic makeup, or a combination of the two, ultimately resulting in a failed healing response of the Achilles Tendon. The genetic makeup of the patients who were included within this scoping review is not available. Further genetic studies would help to delineate the genome associations of EOAT.

### 4.5. Treatment

**Figure 2:**
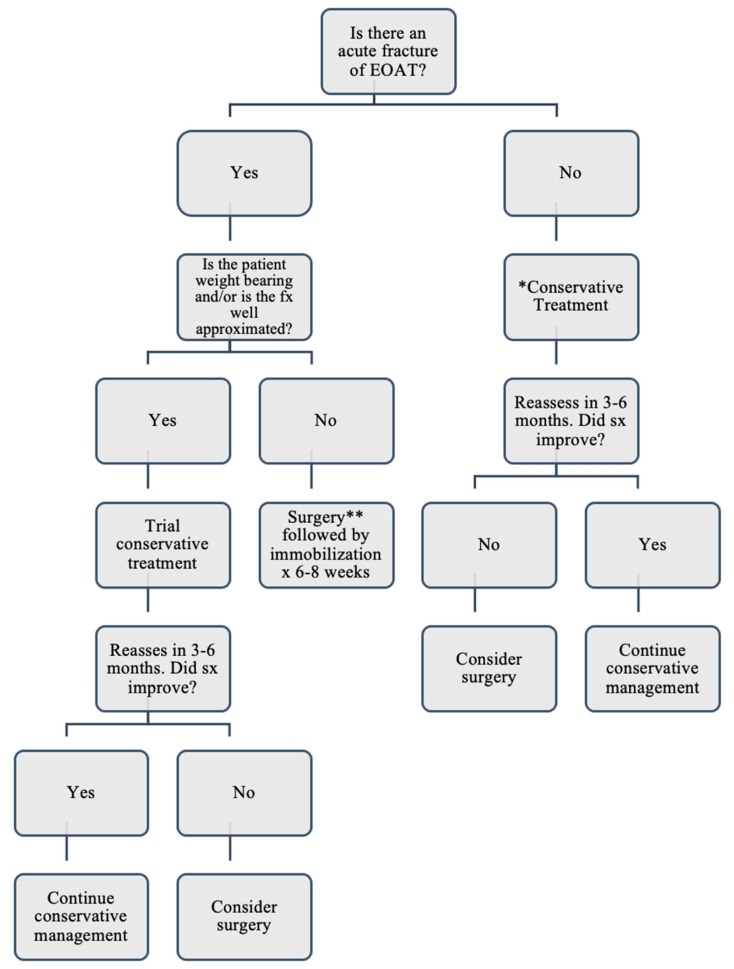
Management of Patients Presenting with an Extensive Ossification of the Achilles Tendon. * Conservative treatment: eccentric exercises, ice, heat, topical diclofenac or other topical agents, oral non-steroidal anti-inflammatory drugs (NSAIDs), and physical therapy. ** Surgery: various methods have achieved the same results of restoring continuity and function of the AT.

Figure 2 provides an algorithm with a reasonable approach to treating EOAT based on the treatment methods in the literature. To determine the most appropriate treatment in a patient with EOAT, medical providers should consider the pattern of onset, functionality of the patient, and the integrity of the Achilles Tendon. Currently, there is insufficient evidence to suggest that patients with Extensive Ossification have better outcomes with surgery than conservative treatment. However, the extent of the ossification has been reported to be one of several determining factors when deciding to treat surgically rather than conservatively. If the patient presents with EOAT that is painful but not fractured, the evidence in this scoping review recommends beginning with conservative treatment, including eccentric exercises, ice, heat, topical diclofenac or other topical agents, oral non-steroidal anti-inflammatory drugs (NSAIDs), and physical therapy [9]. However, if the patient presents with EOAT and an acute fracture of the ossification, the provider should determine whether surgery or conservative management is the best initial option. The physician should consider the severity of the patient’s symptoms, weight bearing ability, and how much of the tendon is intact. If the fracture of the ossification is well approximated and if the patient has mild to moderate pain when weight bearing, has adequate range of motion, and can still perform limited function, beginning with conservative treatment would be an acceptable approach, bearing in mind conservative treatments have not consistently been effective for treating EOAT [5,18]. Surgical excision and reconstruction followed by immobilization is shown to be the treatment of choice for those who have not responded to conservative methods [1,5,18,20,30,37,38]. If conservative treatment does not reduce symptoms within approximately 3–6 months, the provider and patient should consider surgical reconstruction. Finally, if the patient has severe pain and/or cannot bear weight, or if there is complete rupture of the Achilles Tendon, surgical reconstruction as the initial treatment would be appropriate. Although several surgical treatments have been reported, the primary goal of surgical treatment is to preserve the continuity and function of the Achilles Tendon [4,13]. Some instances may require complete removal of the ossification and tendon and replacement with an autograft. Several types of autografts have been reported, including grafts from the semitendinosus, gracilis, fascia lata, tendon of the gastrocnemius-soleus muscle, and flexor hallicus longus tendons [5,12,13,15]. Use of each one of these types of autografts have resulted in successful short-term outcomes in the case reports, often restoring full functionality; however, only one of the reports included outcomes after one year. Therefore, it is reasonable for the surgeon to choose a surgical approach based on personal preference and the extent of the tissue damage until further evidence of this condition suggests an optimal standard approach [13]. Following surgery, the ankle should be immobilized in equinus for six to eight weeks [5,12,13,18,20,28,37], at which point the patient can begin bearing weight.

## 5. Conclusions

This scoping review highlights the proposed pathogenesis, risk factors, and various treatment modalities for EOAT. Due to the rarity of this condition, the existing literature regarding EOAT provides limited information regarding the typical risk factors, presentation, treatments, and outcomes. There is inadequate evidence as to which treatment plan provides the best outcome; however, conservative treatment of EOAT without acute fracture or uncomplicated and well approximated fracture is the preferred initial treatment followed by surgical repair with autograft for complicated fractures of the ossification. Presently, there is no standardization of repair including surgical approach or type of autograft. This leads to difficulty in assessing the efficacy of a standardized treatment plan. Further investigation may include a case-control study or retrospective cohort study to assess the treatments with optimal outcome in each type of presentation. Additionally, because most patients present for evaluation when the ossification is in the later stages there is a lack of evidence regarding the pattern of progression of EOAT. The presence of atraumatic EOAT in genetically related family members suggests there may be a genetic component to pathogenies. Further genetic studies of patients with EOAT should be conducted to identify any alleles or other genetic patterns that are associated with the condition. The challenge of carrying out future studies of EOAT will be the identification of enough individuals with this condition to gather sufficient data in order to make an assessment regarding optimal management. However, we strongly advocate for further evaluation and discussion of the varying treatment options that assesses long-and short-term outcomes of each treatment approach. 

## Figures and Tables

**Table 1:** Relevant articles (Case Reports; 1932–2020) and their main findings.

Author(s), Year	Main Findings
Mallinson, 1932 [18]	A 56-year-old man. Bilateral ossification with fracture of one AT; born with a deformity of both feet (bilateral talipes equinus) with a tenotomy performed at 8 years old. Treated surgically: made an incision up the tendon, drilled holes in the tendon, and used wires to bring the ends of the fracture together.
Brotherton, 1979 [19]	A 71-year-old man. Fracture of Achilles Tendon (AT) with Extensive Ossification; history of soft tissue injury to right calf 15 years prior and subcutaneous tenotomy of the left AT as an infant. Treated by holding the two ends of the fracture in place with a figure eight wire, equinus for 4 weeks, then bore weight in a cast 3 weeks after.
Proctor and Epps, 1976 [20]	A 29-year-old man. Progressive ossification of AT following kick injury to left heel. Initially treated conservatively and placed in walking cast for 4 weeks but pain persisted with thickened heel cord. 10 weeks post injury he had worsening pain with X-ray revealing early ossification. 5 months post injury he had impaired function with continued ossification progression that continued for 2 months until surgical excision. Patient placed in leg cast for 6 weeks. Five months post-op had no tenderness and could run without difficulty.
Weseley, 1976 [21]	A 58-year-old man. Fracture of AT with ossification; history of poliomyelitis as a child with several operations to help him walk better, no history of trauma. Treated by splint for 6 weeks with no tenderness and good healing, but patient had another injury to the tendon month later. Two large fragments were removed and remnants of the Achilles Tendon were repaired. Reconstruction and reinforcement of the tendon was performed using the peroneus brevis tendon.
Fink, 1982 [15]	A 42-year-old woman. Fracture of ossified AT; no history of trauma to tendon; treated surgically. The plantaris tendon was incorporated into the bony mass upon exploration during surgery. Used a tendon graft from the gastrocnemius-soleus muscle with the ossification left in place. Cast with knee in 30 degrees of flexion and in maximal plantarflexion. Immobilized for 11 weeks followed by range of motion and stretching exercises.
Raynor, 1986 [22]	A 57-year-old man. Ossification of AT with intermittent burning pain and swelling over left heel that progressively worsened over one year. No past history of trauma to involved area but did have history of acute gouty arthritis in first metatarsophalangeal joints of both feet. Treated through surgical excision of retrocalcaneal exostosis and ossified deposits within the Achilles Tendon.
Suso, 1988 [23]	A 20-year-old man. Transverse fracture of AT with ossification of almost entire Achilles Tendon. Long-distance runner experienced sudden pain and swelling during athletic competition; history of mild pain in posterior left ankle during sports for previous 2 years. Treated with surgery: the ossification was removed, and reconstruction of the tendon, which did not show loss of continuity, was performed using the Bosworth technique.
Friedman, 1991 [24]	A 41-year-old woman. Fracture of AT with Extensive Ossification by twisting left ankle; history of complete union of left distal tibial fracture one year previously. Due to persistent pain, treatment involved surgical excision of ossific masses.
Banerjee, 1992 [25]	A 79-year-old man. Sharp sudden pain with palpable gap in Achilles and tendons that felt hard, positive squeeze test.Treated with a below-knee-plaster-of-Paris cast in full equinus. Wore cast for 6 weeks, then 2 weeks of a Hartshill splint. Afterward, there was continuity of the Achilles.
Joshi et al., 1994 [26]	A 42-year-old woman. Ossified AT without fracture, with pain; history of AT rupture as a 13-year-old requiring surgical repair; treated conservatively.
Parton et al., 1998 [27]	An 84-year-old man. Ossified Achilles Tendon fracture with examination revealing extensive bilateral AT ossification; history of bilateral surgical repair for clubfoot at 6 years old. Treated conservatively with cast immobilization for 2 months followed by gradual mobilization. Three months after injury the patient was mobile with stiffness and discomfort at site.
Marino, 1999 [28]	A 58-year-old man. Fracture of ossified AT onset suddenly while walking. Patient had taken repeated courses of oral steroids for chronic bronchitis and was taking a short course at the time of injury onset. He had no prior ankle problems and no history of club foot repair. Repair was completed with a modified Kessler suture through bone and tendon. Serial casts were used for 8 weeks, and the patient began weight bearing at 6 weeks. Six months post op the patient was ambulating without pain or difficulty and had 80% active plantarflexion/dorsiflexion which was maintained 1 year post op.
Haddad et al., 1999 [8]	A 67-year-old woman. Avulsion fraction to AT with Extensive Ossification; no history of previous trauma to AT. Non-operative management due to poor skin, obesity, hypertension, and her wish to avoid surgical intervention. Placed in equinus below-knee plaster for 6 weeks with gradually decreasing equinus for 3 additional weeks. Six months after injury the patient was able to walk with aid.
Mady, 2000 [4]	A 57-year-old man. Bilateral AT ossification with fracture on one side; history of bilateral clubfoot treatment with serial plaster casts. Treated surgically followed by short leg cast for 6 weeks. 7 years post-op the patient showed full recovery with complete healing.
Sobel et al., 2002 [29]	A 61-year-old woman. Ossification of a ruptured Achilles with fracture of the ossified mass; history of diabetes mellitus for 15 years. 1 year of conservative treatment consisting of immobilization, ultrasound therapy, stretching and NSAIDs did not relieve pain. The mass was then removed surgically with reattachment of the tendon, followed by casting. 1-year follow-up showed the AT remained indurated but with resolution of symptoms.
Sasaki et al., 2005 [30]	A 51-year-old man. Bilateral ossifications of the AT with dull pain (15mm on left, 55mm on right). Left sided mass surgically removed with consequent reduction in pain, with the right sided mass conservatively treated. At 61 years old, the patient presented again with a 10mm ossification of the left AT proximal to the previous site. The right ossification enlarged to 60mm. Conservative treatment with etidronate disodium tried for 2 years with both ossifications growing in size. The left mass was then excised to relieve pain.
Battaglia, 2006 [5]	A 55-year-old man. Fracture of ossification within AT; history of AT rupture at 25 years prior. Failed non-operative treatment for 3 months, subsequently underwent debridement with reconstruction using the flexor hallucis longus tendon. Serial casting in equinus was completed for 8 weeks. The patient then was placed in a fixed ankle walker for the following 6 weeks. At 14 weeks he had full, painless range of motion. At 6 months follow up, he was prescribed physical therapy. One year post operation the patient had ROM bilaterally and at 18 months had no restrictions.
Richards et al., 2008 [31]	A 53-year-old woman. Peritendinous edema associated with Extensive Ossification of Achilles Tendon; history of injury to right calf with torn Achilles 4 years prior. Patient treated with therapeutic injection followed by other non-operative treatments.
Tamam et al., 2011 [32]	A 41-year-old man. Bilateral ossification to the AT near the calcaneus insertion; no history of direct trauma or previous AT surgery. Patient was managed conservatively. Authors noted repetitive microtrauma to the AT as the cause of the ossifications based on the bilateral presentation.
Ross et al., 2014 [14]	A 57-year-old woman. Unilateral ossification of the AT; patient symptomatic for 10 years with a “popping” sensation. No history of acute trauma, but the patient often participated in running ang yoga. Treated with surgical removal of the ossification and placement of an autograft of the flexor hallucis longus tendon; cast for 6 weeks and began physical therapy 12 weeks after surgery.
Arora, 2015 [9]	A 48-year-old woman. Bilateral AT ossification with no history of trauma or surgery; CT revealed multiple ossified bone fragments within AT bilaterally; diagnosed with Type 2 AT ossification.
Majeed et al., 2015 [17]	A 24-year-old man. Multiple ossifications in the right AT; history of bilateral club foot repair with AT elongation as a child. Ossifications were removed with special care to preserve as much of the AT as possible; 12 weeks post-op the patient had complete resolution of symptoms.
Ishikura et al., 2015 [12]	A 50-year-old woman. Fracture of right ossified AT; history of right ankle dislocation and partial AT rupture in high school, which was poorly managed post manual reduction. The patient had pain and swelling in area for years, but fracture occurred 10 days after starting anti-inflammatories. The ossification was removed and the hamstring tendons, the ipsilateral semitendinosus and gracilis tendons were harvested for AT reconstruction. To the authors’ knowledge, this is the first time hamstring tendons had been used for EOAT repair.
Manfreda et al., 2018 [10]	A 66-year-old man. Spontaneous AT rupture while running with extensive asymptomatic ossification of distal AT; history of surgical release for clubfoot as child. Surgical repair of rupture revealed the Extensive Ossification and subsequent removal of entire ossified portion, followed by tendon reconstruction.

AT: Achilles Tendon; CT: Computed topography; NSAID: Non-steroidal anti-inflammatory drug; FOAT: fractured ossification of Achilles Tendon.

**Table 2:** Relevant articles (Clinical Images; 1990–2020) and their main findings.

Author(s), Year	Main Findings	
Wick and Rieger, 2008 ^a^ [33]	A 69-year-old man. This image submission includes two x-ray images of a complete rupture of a calcified Achilles’ tendon of a 69-year-old male (Images A and B). Arrows indicate the presence of a complete rupture of the calcified Achilles Tendon. The man reported previous partial cutting of his Achilles’ tendon when he was a teenager. The man felt a sudden pain while standing from a seated position, where he proceeded to the emergency room for imaging.	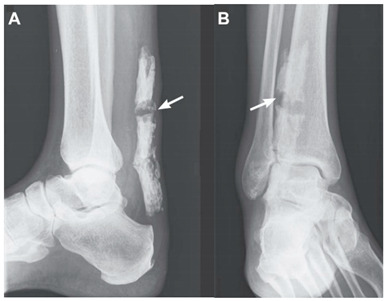
Lin et al., 2012 ^b^ [34]	A 70-year-old woman. This image submission includes two radiographs of a 70-year-old woman (Image C). The patient presented with a sudden stabbing ankle pain. She denied any history of trauma or surgery but did report frequent local injections of her right ankle for pain and soreness. Radiographs demonstrate complete rupture of the calcified Achilles Tendon and a 3 cm separation shown in the first radiograph. Direct suture repair was performed and the follow-up radiograph revealed minimal gap in the tendon (Image D).	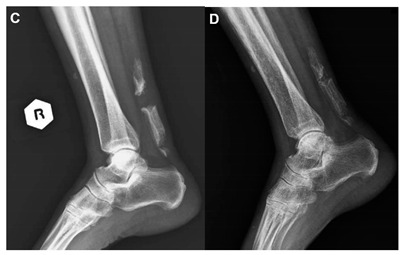
Akhaddar, 2014 ^c^ [7]	A 52-year-old man. This image submission included plain radiographs with a lateral and an antero-posterior view of the right leg and ankle. Arrows indicate a 10-cm ossification of the Achilles Tendon without a fracture (Images E and F). The patient presented after acute trauma to the foot, and the ossified mass was an incidental finding. The patient had no symptoms of the lower leg at presentation and had no history of metabolic or systemic illness nor ankle trauma. Surgery was not performed due to the lack of symptoms.	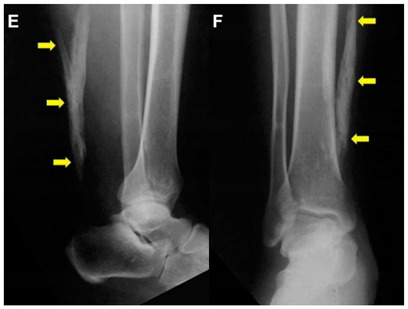
Romano et al., 2020 ^d^ [11]	A 58-year-old woman. This image submission includes a lateral view X-ray (Image G), ultrasound (Image H), MRI (Image I), and STIR image (Image J). In image G the asterisk represents the gap separating the two ossifications (arrows) within the Achilles Tendon. Image H demonstrates a hyperechoic gap (asterisk) between tendon ends (arrows) which is suggestive of a complete rupture of the ossification. Images I and J demonstrate hemorrhage and edema (asterisk) filling the space between the separated ends of the Achilles Tendon. The patient had a palpable gap on examination of the Achilles Tendon. The images confirmed the diagnosis of a complete rupture of ossified tendon. The patient had no history of trauma or surgery in the area. The patient was managed with immobilization in a plaster cast. The patient’s recovery status was not reported.	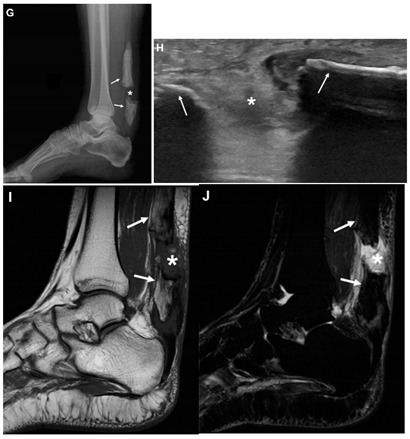
Sullivan and Thurston, 2021 ^e^ [35]	A 72-year-old man. This image submission includes an ultrasound (Image K), lateral view x-ray (Image L), and an MRI (Image M) of a patient who presented to an orthopedic sports medicine clinic after feeling a “pop” while mowing his lawn. Image K is an ultrasound of the Achilles Tendon that depicts a segment of discontinuity (green arrow) within a linear hyperechoic signal. Image L and Image M show ossification of the Achilles Tendon and arrows (red and blue, respectively) indicate fracture of the ossified mass. The patient had no knowledge of the ossification, but he reported having lower leg surgery as a 2-year-old to correct for in-toeing. The patient was treated conservatively with casting and physical therapy. One year after the ossification and coexisting fracture of the ossified mass was discovered, he reported being fully functional without pain.	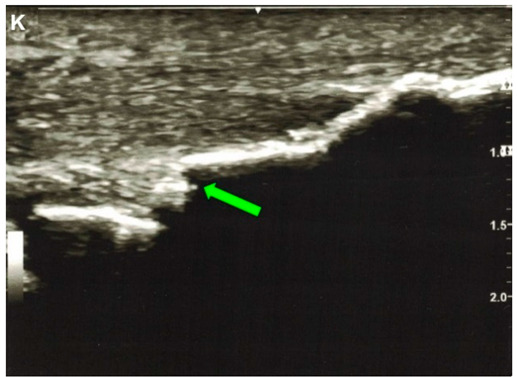 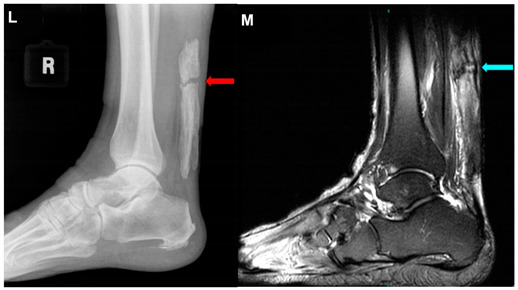

^a^ Images were reproduced with permission from Wick M and Rieger M, The New England Journal of Medicine, 2008. ^b^ Images were reproduced with permission from Lin et al., The American Journal of the Medical Sciences, published by Elsevier, 2012. ^c^ Images were reproduced with permission from Akhaddar A, Pan African Medical Journal. 2014. ^d^ Images were reproduced with permission from Romano N, Diagnostic and Interventional Imaging, 2020. ^e^ Images were reproduced with permission from Sullivan D and Thurston M, Journal of Osteopathic Medicine, published by De Gruyter, 2021.

**Table 3:** Relevant articles (Case Reports and Clinical Images; 1932–2021) and their main findings.

Author(s), Year	Age (years)	Male	Female	Fracture of Ossification	Treated Conservatively	Treated Surgically	History of Trauma
Brotherton, 1979 [19]	71	✓		✓		✓	✓
Mallinson, 1932 [18]	56	✓		✓		✓	✓
Proctor and Epps, 1976 [20]	29	✓				✓	✓
Weseley, 1976 [21]	58	✓		✓	✓	✓	✓
Fink and Corn, 1982 [15]	42		✓	✓	✓		
Raynor, 1986 [22]	57	✓				✓	
Suso, 1988 [23]	20	✓		✓		✓	✓
Friedman, 1991 [24]	41		✓	✓		✓	✓
Banerjee, 1992 [25]	79	✓		✓	✓		
Joshi et al., 1994 [26]	42		✓		✓		✓
Parton et al., 1998 [27]	84	✓		✓	✓		✓
Marino, 1999 [28]	58	✓		✓		✓	
Haddad et al., 1999 [8]	67		✓		✓		
Mady and Vajda, 2000 [4]	57	✓		✓		✓	✓
Sobel et al., 2002 [29]	61		✓	✓		✓	
Sasaki et al., 2005 [30]	51	✓			✓	✓	
Battaglia and Chandler, 2006 [5]	55	✓		✓		✓	✓
Richards et al., 2008 [31]	53		✓		✓		✓
Wick & Rieger, 2008 [33]	69	✓		✓	N/A	N/A	✓
Tamam et al., 2011 [32]	41	✓			✓		
Lin et al., 2012 [34]	70		✓	✓		✓	
Akhaddar, 2014 [7]	52	✓			✓		
Ross et al., 2014 [14]	57		✓	✓		✓	
Arora, 2015 [9]	48		✓		N/A		
Majeed et al., 2015 [17]	24	✓				✓	✓
Ishikura et al., 2015 [12]	50		✓	✓		✓	✓
Manfreda et al., 2018 [10]	66	✓				✓	✓
Gendera et al., 2020 [13]	70	✓		✓		✓	
Romano et al., 2020 [11]	58		✓	✓	✓		
Sullivan & Thurston, 2021 [35]	72	✓		✓	✓		✓
Totals Percentage		19/30 (63%)	10/30 (33%)	18/30 (60%)	12/30 (40%)	17/30 (56%)	16/30 (53%)

AT: Achilles Tendon.

**Table 4:** Relevant articles (Case Series; 1994–2013) and their main findings.

Author(s), Year	Main Findings
Yu et al., 1994 [2]	16 ossified ATs from 12 patient were imaged to characterize the imaging abnormalities associated with AT ossification, with the most constant finding being thickening of the tendon at the site of ossification. The authors also noted that ossification occurred in the substance of the tendon in 8 tendons, while in 8 tendons ossification occurred within 2 cm of insertion.
Hatori et al., 2002 [36]	Three men who all had atraumatic ossification of bilateral AT with no history of direct trauma, surgery, or club foot release. The patients with pain underwent surgical removal ossification, and the one patient without pain did not undergo surgical correction. All symptoms improved with treatment. Ossification in the AT was found to be a product of both endochondral and intramembranous ossification and was likely to be the result of repetitive microtraumas.
Cortbaoui et al., 2013 [37]	Three siblings (50-year-old female, 64-year-old female, 54-year-old male), all previously healthy, with ossification of the Achilles Tendon (OAT), suggesting a possible genetic predisposition of OAT.
Howell et al., 2019 [38]	Retrospectively reviewed the outcomes of patient-reported satisfaction in 45 patients with past surgical management of calcific insertional Achilles Tendinopathy. The success rate with nonsurgical treatment decreases when the patient has tendinosis changes as well. The study suggested that surgical treatment should be considered after 3–6 months of failed conservative therapy.

AT: Achilles Tendon; OAT: ossification of the Achilles Tendon.

**Table 5:** Relevant articles (Molecular Studies; 2004–2019) and their main findings.

Author(s), Year	Main Findings
Hannallah et al., 2004 [39]	The delivery of Noggin mediated by muscle-derived stem cells can inhibit heterotopic ossification caused by BMP-4, demineralized bone matrix, and trauma in an animal model. Used a retroviral vector and muscle-derived stem cells to deliver Noggin to mouse model.
Lin et al., 2010 [40]	Rat model for heterotopic ossification (HO) induced by Achilles tenotomy evaluated for mRNA and protein expression levels at 3, 5, and 10 weeks. HO induced by Achilles tenotomy is by endochondral bone formation, and HIF-1 alpha activation plays an important role during chondrogenesis in this model.
Shimono et al., 2011 [41]	Displayed inhibition of HO through the use of RARγ agonists targeting the BMP-2 Smad pathway in standard muscle trauma-associated HO mouse model.
O’Brien et al., 2013 [42]	20-week long study in mice evaluating the biomechanical responses of mineralized tendons and effect of mineralization on contralateral tendon through the murine needle injury model. The study found that the mean volume of tendon mineralization in the contralateral limbs of injured mice was three times greater compared with the limbs from the controls.
Peterson et al., 2015 [43]	The authors describe a burn/tenotomy mouse model to produce HO in predictable locations.
Agarwal et al., 2016 [44]	Focuses on the cells involved in HO formation (bone–cartilage–stromal progenitor cells). Mouse model of HO (trauma and genetic models) and evaluation of human samples. Adventitial cells, but not pericytes, appear to play a supportive role in HO formation. Findings indicated that, rarely, BCSPs are derived from a systemic source outside of the local tissue environment.
Zhang et al., 2016 [45]	Injury-induced ectopic tendon mineralization is progressive by 10-week and 25-week samples. In vitro mechanical testing showed that max force, max stress and mid-substance modulus in the 25-weeks samples were significantly lower than the 10-weeks samples. Treatment with the BMP receptor kinase inhibitor significantly inhibited injury-induced tendon mineralization. Phosphorylation of both Smad1 and Smad3 were highly increased in injured tendons as early as 1-week post-injury and remained high in ectopic chondrogenic lesions 4 weeks post-injury.
Wang et al., 2018 [46]	TGF-β inhibition through the use of both antibodies and gene knockout prevents HO progression.
Tuzmen et al., 2018 [47]	Evaluated the effects of neuropeptides substance P and calcitonin gene-related peptide on heterotopic ossification in murine model in Achilles Tendons. Substance P caused HO and calcitonin gene-related peptide inhibited HO formation by Substance P.
Chen et al., 2019 [48]	The use of curcumin in rodent tendon ectopic calcification models partially rescued tendon calcification.

BMP: Bone Morphogenic Protein; HO: heterotopic ossification; HIF: hypoxia inducible transcription factor; RARγ: retinoic acid receptor γ; BCSP: Bone—cartilage—stromal—progenitor cells; TGF-B: Transcription Growth Factor-β.

**Table 6:** Relevant articles (Clinical Studies; 2007, 2017) and their main findings.

Author(s), Year	Main Findings
Ateschrang et al., 2007 [49]	Retrospective study that analyzed post-op AT calcification after open-augmented repair on 104 patients with Achilles Tendon rupture. Incidence of tendon calcification was 14.4%, with the low incidence attributed to the NSAID diclofenac and its anti-calcifying effects.
Ateschrang et al., 2017 [50]	Analyzed the risk factors and potential clinical impact of postoperative tendon calcification after percutaneous Achilles Tendon repair in 126 patients with an acute, complete Achilles Tendon rupture, with 81 patients available for follow-up. PTC incidence was 11.1% (9 patients), with male gender and advanced age being risk factors. The authors postulated that the use of NSAIDs aided in the low incidence rates.

AT: Achilles Tendon; NSAID: non-steroidal anti-inflammatory drug; PTC: postoperative tendon calcification.

**Table 7:** Relevant articles (Reviews; 1990–2020) and their main findings.

Author(s), Year	Main Findings
Garland, 1990 [51]	Author discusses the commonalities in the clinical courses of HO including the most common signs and symptoms, treatment modalities, and surgical timing.
Dobbs and Burnett, 2009 [52]	Provides an update on clubfoot etiology and current treatment strategies in order to aid in determining prognosis and the selection of appropriate treatment. A tenotomy of the AT is commonly performed with casting for the correction of clubfoot.
Oliva et al., 2012 [3]	The authors undergo a review of Calcific Tendinopathy (CT) in order to inform future treatment modalities, while advising for the use of ‘Calcific Tendinopathy’ and ‘insertional Calcific Tendinopathy’ to distinguish between different pathogenic mechanisms.
O’Brien et al., 2012 [6]	Reported 15% of cases following repair of the Achilles Tendon have mineralization with no negative effects on clinical outcome as measured by the Thermann score. Endochondral ossification (most frequent) and Intramembranous ossification are both thought to happen in the human Achilles Tendon. BMP signaling seems to play a role in heterotopic ossification, while hypoxia and hypoxia-inducible transcription factor (HIF) signaling critical to EO process.
Hwang, 2018 [53]	By systematically using the distribution pattern of the calcified lesions, such as ossification of the Achilles Tendon, shown in this review, additional laboratory testing, and clinical history, appropriate differential diagnoses can be approached.
Jukes et al., 2019 [54]	Focuses on the diagnosis and management of posterior heel pain including Achilles Tendinopathy, both insertional and non-insertional; provides treatment recommendations for symptoms of varying lengths.
Maffulli et al., 2020 [1]	Discusses options for treatment of Achilles Tendinopathy. Defines tendinopathy as a failed healing response with changes in collagen fibers caused by a combination of intrinsic and extrinsic factors. Recommends that patients be treated with non-operative care for a minimum of 3–6 months before undergoing surgery.
Zhang, 2020 [55]	A variety of signal pathways involve heterotopic ossification of tendon and ligament (HOTL) with multiple roles in different stages of heterotopic ossification formation. The review summarizes the progress and provides an up-to-date understanding of HOTL.

AT: Achilles Tendon; CT: Calcific Tendinopathy; BMP: Bone Morphogenic Protein; HIF: hypoxia inducible transcription factor; HOTL: heterotopic ossification of tendon and ligament; EO: Extensive Ossification.
